# An analysis of risk factors and current status of depressive mood in mid-to-late adolescence and early adulthood in northern China

**DOI:** 10.3389/fpsyt.2024.1370601

**Published:** 2024-07-04

**Authors:** Xueping Yang, Junxiao Miao, Yunlong Bai, Lili Li, Gengsen Zhuang

**Affiliations:** ^1^ Department of Psychology, The People’s Hospital of Liaoning Province, Shenyang, China; ^2^ Department of Information Technology, The 20th Middle School of Shenyang, Shenyang, China; ^3^ Department of Psychiatry, The First Affiliated Hospital of China Medical University, Shenyang, China; ^4^ Department of Psychiatry and Mental Health, The Medical University of Dalian, Dalian, China

**Keywords:** depression, anxiety, self-injury, adolescence, early adulthood, China

## Abstract

**Introduction:**

At present, the incidence of adolescent depression is increasing each year, having a wide and profound impact on adolescents. This study investigated the mood state of mid-to-late adolescents and young adults and analyzed related factors; clarified the incidence of depression, suicide, and self-injurious thoughts/behaviors in this population; and conducted relevant analysis of related factors of depression and anxiety.

**Methods:**

Study subjects were students aged 14–25 years, from three high schools and one university in Liaoning Province. Study subjects were evaluated using several questionnaires that combined online and offline methods. Specifically, the Self-rating Depression Scale (SDS), Self-rating Anxiety Scale (SAS), Simplified Coping Style Questionnaire (SCSQ), Child Depression Inventory (CDI), the Chinese version of the Spence Child Anxiety Scale (SCAS), and a general questionnaire were utilized. Single-factor ANOVA, t-test, Chi-square, and multiple regression analysis were used to analyze the data.

**Results:**

The results showed that, among the 14–17-year-old subjects, the incidence of depression was 336 (14.7%), the incidence of anxiety was 763 (33.5%). Among the 18–25-year-old subjects, the incidence of depression was 34 (8.6%), the incidence of anxiety was 7 (1.8%). In the general questionnaire, 2081 (77.8%) individuals were depressed, 689 (25.8%) had thoughts of self-injury, and 323 (12.1%) had self-injurious behaviors. Among the 14–17-year-old subjects, 1789 (78.46%) were depressed, 689 (30.22%) had self-injury thoughts, and 319 (1.71%) had self-injurious behaviors. Among the 18–25-year-old subjects, 292 (73.92%) were depressed, but 4 (1.01%) had self-injurious behaviors. The incidence of depression and anxiety in adolescents is high, presenting with a certain risk of self-injury. However, age is an important factor in the occurrence of depression and anxiety; among the 18–25-year-old subjects, the incidence of depression (8.6% vs. 4.7%) and anxiety (1.8% vs. 33.5%) was lower than that among the 14–17-year-old population. Through comparative analysis, adolescents aged 14–17 remained at high risk of depression and anxiety.

**Discussion:**

In the analysis of risk factors for depression and anxiety, relationships with classmates, teachers, and parents were reported as important influencing factors of emotional state. Further, a good coping style was found to be protective against depression and anxiety.

## Introduction

The incidence of depression among adolescents is increasing each year, which is not only a significant social problem but also a major public health problem. Depression is the leading cause of morbidity and disability globally. Depression can also have a wide and profound impact on the adolescents, especially in cases of self-injury and suicide. Further, depression can cause various problems, such as school boredom, social avoidance, violence, extreme behavior, and suicide. If not treated on time, even if it is not life-threatening, depressive symptoms are likely to continue to adulthood, affecting family happiness and social stability (1, 2). Recent studies have reported that adolescents exhibit a high incidence of depression. Depression commonly onsets in adolescence, affecting approximately 1 in 4 female adolescents and 1 in 10 male adolescents in the United States. Adolescent depression is a significant risk factor for suicide, causing over a third of all American adolescent deaths. Adolescent depression is typically discussed alongside its developmental and gendered considerations with a focus on important risk factors, including non-suicidal self-injury, adverse childhood experiences (ACEs), and substance abuse (3). The COVID-19 pandemic and its accompanying infection control measures introduced sudden and significant disruptions to the lives of children and adolescents around the world. A systematic review and meta-analysis to examine the prevalence of depressive symptoms, anxiety symptoms, and sleep disturbances in children and adolescents ≤ 18 years of age during the pandemic, were reported at 31%, 31%, and 42%, respectively. Age, grade level, education level, gender, geographical region, and electronics use were correlated with the prevalence of mental health symptoms (4).

Studies have shown that the diagnosis of mental disorders, including mood/anxiety disorders in childhood or adolescence, is closely related to an increased risk of suicidal behavior and adverse adult social outcomes in adulthood. Adolescent suicide attempts increase the likelihood of suicide deaths and suicide attempts in adulthood, as well as affect other aspects, such as academic studies and adult criminal offenses (5). Therefore, adolescent emotional problems have an important impact on the psychological status of adults. Self-injury and suicide are important manifestations for adolescent emotional problems. Death caused by suicide is the most serious adverse consequence of depression. In the adolescent population, individuals in the depressed phase tend to seriously consider suicide. There is a strong relationship between suicide attempt and death by suicide. Moreover, non-fatal forms of suicide and self-injury have become the most common causes of hospitalization among adolescents in many countries in the field of psychiatry (6).

However, it is very important to understand how depression and bad mood occur in adolescents, and many studies have been conducted about the causes of emotional problems in adolescents and early adulthood. Stefania Maccari et al. found early adverse experiences, including mother-pup interactions, shape the response of an individual to chronic stress or to stress-related diseases during adult life in mammals, by summarizing the findings of previous animal models and humans studies. It pointed out the importance of programming during the early developmental period even including postpartum, after birth or during the first year of life (7). The production of psychiatric illness is associated with an earlier pathology. From the family aspect, parental marital discord, poor communication, and parenting behavior imbalance are associated with the occurrence of depression in offspring-adolescent depression. At the level of the individual, differences in individual reactions, behaviors, cognitive characteristics of negative attribution, temperament characteristics, personal vulnerability, emotional instability, poor self-esteem, and low social ability are also associated with an increased risk and severity of depression. Recent studies have also shown that the occurrence of adolescent depression may be related to parenting style, mobile phone addiction, personality dysfunction, psychological disorders within the individual, and congenital factors (8, 9). In China, studies have also reported risk factors related to adolescent depression, suicidal intentions, and self-injury behaviors, such as parenting styles, especially negative parenting styles (10, 11). Additional research has shown that negative coping styles, childhood traumatic experiences (12), and adolescent interpersonal relationships (13) can also affect adolescent depression. Furthermore, research has shown that the interaction and cumulative effects of ACEs, such as verbal violence, poor interpersonal relationships, and even divorce/separation, significantly increase the probability of suicide in early adulthood and adolescence in all individuals, with females especially affected. The effect of ACEs on the incidence of suicide outcomes was significant (14). In general, the occurrence of adolescent depression, self-injury, and suicide is related to many factors.

Therefore, previous epidemiological studies have found a high proportion of adolescent depression, summarized the factors of adolescent depression, and then proposed measurement tools, prevention programs, and different types of clinical treatment for adolescent depression. However, it has also been suggested that the early diagnosis model should be adjusted and prevention programs should be developed and implemented as soon as possible (15). Research and practice have focused more on effective interventions, rather than on prevention and early identification; thus, more relevant research data are needed for prevention and early warning identification. As such, the current study sought to acquire and improve relevant data on this subject. This study investigated depressive mood and analyzed related factors in mid-to-late adolescence and early adulthood; clarified the incidence of depression, suicide, and self-injurious thoughts and behaviors in adolescence and early adulthood; and conducted investigation and analysis of anxiety and related factors, to determine relevant factors that may affect the occurrence of depression, self-injury, suicide, and anxiety.

## Methods

### Subjects

Subjects were students 14–25 years of age selected for the study from three high schools and one university in Liaoning Province. Exclusion criteria included developmental delay, severe mental disorders, severe physical diseases, and/or other developmental abnormalities. All participants and/or their guardians provided informed consent and privacy was protected. The study protocol was approved by the Ethics Committee of the People’s Hospital of Liaoning Province.

### Research tools

Research subjects were evaluated using the following questionnaires, both online and offline: the Self-rating Depression Scale (SDS, standardized score ≥ 50 points indicating depressive symptoms); the Self-rating Anxiety Scale (SAS, standardized score ≥ 50 points indicating anxiety symptoms), (Zung, 1965) (16, 17); the Simplified Coping Style Questionnaire (SCSQ, a coping propensity value > 0 indicates that the study subject mainly adopts a positive coping style in the stress state, and a coping propensity value < 0 indicates that the study subject mainly adopts a negative coping style in the stress state) (18); the Child Depression Inventory (CDI, scores ≥ 19 indicate depressive symptoms for adolescents aged 14–17 years) (19); the Chinese version of the Spence Child Anxiety Scale (SCAS, total score ≥ 25 indicates anxiety for participants 14–17 years of age); and a general questionnaire that included various elements, such as characteristics of family structure, stress environment, Internet preferences, physical exercise, and sleep satisfaction. These questionnaires were distributed in the form of online small program and paper version, respectively. Paper version was mainly used in the population of middle school and high school students, and online small program was mainly used in the population of college students. After the completion of the questionnaires, the data was collected and sorted out.

### Statistical analysis

SPSS statistical software (version 22.0, SPSS Inc., Chicago, IL, USA) was used to analyze all data. Single-factor ANOVA, T-test, Chi-square, and multiple regression were used. The results are expressed as frequency, percentage (%), and mean (± SD). P < 0.05 indicated a significant difference, and P < 0.01 indicated a highly significant difference.

## Results

A total of 2675 valid questionnaires with complete data were received online and offline, representing an effective return rate of 98.3%. Among the valid study subjects, 1195 (44.7%) were males and 1480 (55.3%) were females. Ages ranged from 14 to 25 years (mean age: 16.18 ± 1.34 years), of which 2280 study subjects (85.2%) were aged 14–17 (mean: 15.77 ± 0.85 years) and 395 study subjects (14.8%) were aged 18–25 (mean: 18.56 ± 1.14 years). General demographic information and responses to the general questionnaire are shown in **Table 1**.

**Table 1:** Survey of sociodemographic characteristics and general conditions of participants.

Variables		N (%)
Gender	Male Female	1195 (44.7%) 1480 (55.3%)
Age	14–17 years 18–25 years	2280 (85.23%) 395 (14.77%)
Family composition	Nuclear family (children and parents) Single parent family Reconstituted Family Large family: 3 generations	1959 (73.2%) 135 (5.0%) 44 (1.6%) 537 (20.1%)
Are you an only child?	Yes No	1997 (74.7%) 678 (25.3%)
Do you feel like your parents (caregivers/elders) care enough about you?	Yes No	2584 (96.6%) 91 (3.4%)
Does your family struggle financially?	Yes No	323 (12.1%) 2352 (87.9%)
Are you satisfied with the current state of your life?	Satisfied General Dissatisfied	1842 (68.9%) 737 (27.6%) 96 (3.6%)
Do you regularly participate in sports?	Often Occasionally Hardly	1071 (40.0%) 1222 (45.7%) 382 (14.3%)
Do you enjoy online activities such as gaming or online dating?	Yes No	1558 (58.2%) 1117 (41.8%)
Do you spend a large amount of time on the Internet each day?	Yes No	799 (29.9%) 1876 (70.1%)
Are you getting enough sleep?	Sufficient Insufficient Seriously insufficient	1071 (40.0%) 1336 (49.9%) 268 (10.0%)
Have you ever been depressed?	Yes No	2081 (77.8%) 14–17 years: 1789 (78.46%) 18–25 years: 292 (73.92%) 594 (22.2%) 14–17 years: 491 (21.54%) 18–25 years: 103 (26.08%)
Have you ever had thoughts of hurting yourself? (thoughts of self-injury)	Yes No	689 (25.8%) 14–17 years: 689 (30.22%) 18–25 years: 0 1986 (74.2%) 14–17 years: 1591 (69.78%) 18–25 years: 395 (100%)
Have you ever done anything to hurt yourself? (behaviors of self-injury)	Yes No	323 (12.1%) 14–17 years: 319 (1.71%) 18–25 years: 4 (1.01%) 2352 (87.9%) 14–17 years: 1961 (98.29%) 18–25 years: 391 (98.99%)
Coping styles	Neutral Positive Negative	62 (2.3%) 2493 (93.2%) 120 (4.5%)
Criteria for depression met	Yes No	14–17 years: 336 (14.7%) 18–25 years: 34 (8.6%) 14–17 years: 1944 (85.3%) 18–25 years: 361 (91.4%)
Criteria for anxiety met	Yes No	14–17 years: 763 (33.5%) 18–25 years: 7 (1.8%) 14–17 years: 1517 (66.5%) 18–25 years: 388 (98.2%)

According to the depression and anxiety scales used, among the subjects who were 14–17 years of age, the incidence rates of depression, anxiety, and comorbid depression/anxiety were 336 (14.7%), 763 (33.5%), and 273 (11.97%), respectively. Among subjects who were 18–25 years of age, the incidence rates of depression, anxiety, and comorbid depression/anxiety were 34 (8.6%), 7 (1.8%), and 4 (1.01%), respectively. However, in the self-reported general survey of the overall sample, 2081 subjects (77.8%) reported depression at some point of their life, 689 subjects (25.8%) reported thoughts of self-injury, and 323 subjects (12.1%) reported self-injurious behaviors. In the 14–17-year-old group, 1789 subjects (78.46%) reported depression at some point of their life, 689 subjects (30.22%) reported thoughts of self-injury, and 319 subjects (1.71%) reported engaging in self-injurious behaviors. Among the subjects who were 18–25 years old, 292 (73.92%) reported depression at some point of their life and 4 (1.01%) reported engaging in self-injurious behaviors (**Table 1**).

In the T-test/ANOVA performed in the 14–17-year-old group, several factors were significantly associated with anxiety, including different family composition (F = 4.451, P = 0.004), feeling cared for enough by parents and elders (F = 9.812, P = 0.002), regular participation in sports (F = 7.346, P = 0.001), adequate sleep (F = 21.632, P < 0.001), depressed mood (F = 20.594, P = 0.000), self-injurious thoughts (F = 19.797, P < 0.001), coping style (F = 33.570, P < 0.001), and interpersonal relationships, such as those with classmates, parents, and teachers (F = 28.831, P < 0.001). Additionally, several factors were also found to have statistically significant associations with depression, such as gender (F = 5.015, P = 0.025), different family composition (F = 7.632, P < 0.001), feeling cared for enough by parents and elders (F = 8.226, P = 0.004), regular participation in sports (F = 12.140, P = 0.030), a large amount of time spend on the Internet (F = 11.161, P = 0.001), adequate sleep (F = 62.124, P < 0.001), depressed mood (F = 66.401, P <0.001), thoughts of self-injury (F = 28.490, P < 0.001), different coping styles (F = 64.440, P < 0.001), and interpersonal relationships, such as those with classmates, parents, and teachers (F = 81.513, P < 0.001). These factors may also be potential factors affecting depression scores (**Table 2**).

**Table 2:** T-test/ANOVA of anxiety and depression scores in participants aged 14–17 years.

Variables N (%)			The anxiety score of participants aged 14–17 years			The depression score of participants aged 14–17 years		
			(X ± S)	T/F	P	(X ± S)	T/F	P
Gender	Male Female	1101 (48.29%) 1179 (51.71%)	20.02 ± 14.92 19.69 ± 13.90	0.290	0.590	12.12 ± 7.63 11.44 ± 6.95	5.015	**0.025**
Age	14 years 15 years 16 years 17 years	90 (3.95%) 889 (38.99%) 768 (33.68%) 533 (23.38%)	20.20 ± 15.12 19.74 ± 14.97 19.21 ± 13.75 20.88 ± 14.20	1.458	0.224	11.44 ± 7.91 12.07 ± 7.59 11.51 ± 7.03 11.70 ± 7.07	0.889	0.446
Family composition	Nuclear family (children and parents) Single parent family Reconstituted family Large family: 3 generations	1698 (74.47%) 99 (4.34%) 33 (1.45%) 450 (19.74%)	20.31 ± 14.61 20.78 ± 15.55 22.49 ± 15.23 17.70 ± ±13.05	4.451	**0.004**	12.03 ± 7.41 13.33 ± 7.69 12.33 ± 7.43 10.40 ± 6.57	7.632	**< 0.001**
Are you an only child?	Yes No	1784 (78.25%) 496 (21.75%)	19.99 ± 14.30 19.32 ± 14.73	0.849	0.357	11.92 ± 7.40 11.23 ± 6.87	3.490	0.062
Do you feel like your parents (or caregivers/elders) care enough about you?	Yes No	2203 (96.62%) 77 (3.38%)	20.02 ± 14.46 14.81 ± 11.32	9.812	**0.002**	11.85 ± 7.34 9.43 ± 5.50	8.226	**0.004**
Does your family struggle financially?	Yes No	244 (10.70%) 2036 (89.30%)	19.63 ± 14.78 19.87 ± 14.35	0.062	0.804	11.68 ± 6.84 11.78 ± 7.35	0.044	0.834
Are you satisfied with the current state of your life?	Satisfied General Dissatisfied	1600 (70.18%) 599 (26.27%) 81 (3.55%)	19.99 ± 14.70 19.50 ± 13.46 19.56 ± 15.23	0.268	0.765	12.01 ± 7.53 11.21 ± 6.73 11.11 ± 6.31	2.943	0.053
Do you regularly participate in sports?	Often Occasionally Hardly	984 (43.16%) 1012 (44.38%) 284 (12.46%)	21.11 ± 15.01 18.64 ± 13.78 19.79 ± 14.08	7.346	**0.001**	12.59 ± 7.94 10.99 ± 6.68 11.69 ± 6.73	12.140	**0.030**
Do you enjoy online activities such as gaming or online dating?	Yes No	1332 (58.42%) 948 (41.58%)	20.05 ± 14.45 19.57 ± 14.32	0.618	0.432	11.97 ± 7.18 11.49 ± 7.45	2.400	0.122
Do you spend a large amount of time on the Internet each day?	Yes No	567 (24.87%) 1713 (71.13%)	18.89 ± 13.95 20.16 ± 14.53	3.311	0.069	10.88 ± 6.87 12.06 ± 7.41	11.161	**0.001**
Are you getting enough sleep?	Sufficient Insufficient Seriously insufficient	849 (37.24%) 1190 (52.19%) 241 (10.57%)	22.24 ± 16.06 18.04 ± 13.27 20.32 ± 12.17	21.632	**< 0.001**	13.86 ± 8.39 10.30 ± 6.32 11.67 ± 5.71	62.124	**< 0.001**
Have you ever been depressed?	Yes No	1789 (78.46%) 491 (21.54%)	19.13 ± 14.10 22.45 ± 15.16	20.594	**< 0.001**	11.13 ± 6.88 14.11 ± 8.23	66.401	**< 0.001**
Have you ever had thoughts of hurting yourself? (thoughts of self-injury)	Yes No	689 (30.22%) 1591 (69.78%)	17.82 ± 14.01 20.73 ± 14.48	19.797	**< 0.001**	10.54 ± 6.85 12.30 ± 7.42	28.490	**< 0.001**
Have you ever done anything to hurt yourself? (behaviors of self-injury)	Yes No	319 (13.99%) 1961 (86.01%)	18.75 ± 13.53 20.03 ± 14.53	2.171	0.141	11.27 ± 6.77 11.85 ± 7.38	1.738	0.188
Coping styles	Neutral Positive Negative	62 (2.72%) 2115 (92.76%) 103 (4.52%)	22.08 ± 18.87 19.25 ± 13.83 30.85 ± 17.95	33.570	**< 0.001**	17.42 ± 9.55 11.30 ± 6.93 18.04 ± 8.66	64.440	**< 0.001**
Are you affected by interpersonal relationships, such as those with classmates, parents, or teachers?	Yes No	1867 (81.89%) 413 (18.11%)	19.09 ± 14.13 23.27 ± 15.08	28.831	**< 0.001**	11.13 ± 6.92 14.65 ± 8.21	81.513	**< 0.001**

P<0.05 (bold values) indicated a significant single-factor-correlation, and P<0.01 (bold values) indicated a highly significant single-factor-correlation for anxiety score and depression score.

In the analyses of depression-related factors, while different from the predicted factors but still within the univariate analysis, 10 factors were found to be related to depression scale score (gender, different family composition, whether cared for enough by parents and elders, regular participation in sports, Internet time, sleep duration, depression, self-injurious thoughts, different coping styles, and being affected by classmates, parents, teachers, and others). The P values were all less than 0.05. However, in further multiple regression analysis, age and gender were used as covariates to analyze the influencing factors of depression, and it was found that age (P < 0.001, OR 0.661, 95% CI 0.537–0.813) was a protective factor. The incidence of depression was lower as age increased. The nuclear family composition (children and parents) was significantly associated with depression (P = 0.047, OR 1.411, 95% CI 1.004–1.983), while feeling sleep level was sufficient was significantly associated with depression (P = 0.005, OR 1.948, 95% CI 1.227–3.093), but neither were protective factors for depression. Unlike the predictors, the proportion of depression in people with self-injury thoughts was not higher than that in the non-self-injury group, and self-injury thoughts were not a predictor of depression (P < 0.001, OR 0.345, 95% CI 0.230–0.519). Regular participation in sports was associated with depression. Participating in sports “Occasionally” was a protective factor for depression (P = 0.008, OR 0.576, 95% CI 0.383–0.868). For coping styles, neutral and positive coping styles were protective factors for depression (P = 0.009, OR 0.388, 95% CI 0.190–0.793; P < 0.001, OR 0.256, 95% CI 0.164–0.401, respectively; **Table 3**). Feeling of adequate sleep time, relationship factors, coping style, self-injurious thoughts, and family composition can gradually become a predictive model of depression, and the difference is significant (all Ps less than 0.05; **Table 4**).

**Table 3:** Multivariate regression analysis of depression in participants aged 14–17 years.

Intergroup analysis of depression in the 14–17-year-old group		Depressed	Non-depressed	df	Distinctiveness	Exp(B)	95% CI	
		N (%)	N (%)				low	up
1.0	intercept			1	0.001			
	@1. Gender: Male Female	176 (15.99%) 160 (13.57%)	925 (84.01%) 1019 (86.43%)	1	0.814	0.970	0.752	1.251
	@2.Age: 14 years 15 years 16 years 17 years	16 (17.78%) 150 (16.87%) 103 (13.41%) 67 (12.57)	74 (82.22%) 739 (83.13%) 665 (86.59%) 466 (87.43%)	1	**< 0.001**	0.661	0.537	0.813
	[@3. Family composition = Nuclear family (children and parents)]	270 (15.91%)	1428 (84.09%)	1	**0.047**	1.411	1.004	1.983
	[@3. Family composition = Single parent family]	15 (15.15%)	84 (84.85%)	1	0.263	1.453	0.755	2.796
	[@3. Family composition = Reconstituted family]	3 (9.09%)	30 (90.91%)	1	0.679	0.762	0.211	2.757
	[@3. Family composition = Large family: 3 generations]	48 (10.67%)	402 (89.33%)	0	. NA	. NA	. NA	. NA
	[@4. Feeling parents or elders (caregivers) caring about yourself enough? = Yes]	330 (14.98%)	1873 (85.02%)	1	0.415	1.438	0.601	3.442
	[@4. Feeling parents or elders (caregivers) caring about yourself enough? = No]	6 (7.79%)	71 (92.21%)	0	. NA	. NA	. NA	. NA
	[@5. Regular participation in sports = Often]	181 (18.39%)	803 (81.61%)	1	0.396	0.839	0.559	1.259
	[@5. Regular participation in sports? = Occasionally]	114 (11.26%)	898 (88.74%)	1	**0.008**	0.576	0.383	0.868
	[@5. Regular participation in sports? = Hardly]	41 (14.44%)	243 (85.56%)	0	. NA	. NA	. NA	. NA
	[@6. Long duration on the Internet every day? = Yes]	58 (10.23%)	509 (89.77%)	1	**0.008**	0.643	0.464	0.892
	[@6. Long duration on the Internet every day? = No]	278 (16.23%)	1435 (83.77%)	0	. NA	. NA	. NA	. NA
	[@7. Getting enough sleep? = Sufficient]	203 (23.91%)	646 (76.09%)	1	**0.005**	1.948	1.227	3.093
	[@7. Getting enough sleep? = Insufficient]	105 (8.82%)	1085 (91.18%)	1	0.325	0.796	0.505	1.254
	[@7. Getting enough sleep?= Seriously insufficient]	28 (11.62%)	213 (88.38%)	0	NA	. NA	. NA	. NA
	[@8. Have ever been depressed? = Yes]	222 (12.41%)	1567 (87.59%)	1	0.232	1.462	0.784	2.725
	[@8. Have ever been depressed? = No]	114 (23.22%)	377 (76.78%)	0	NA	. NA	. NA	. NA
	[@9 Ever had the idea of hurting yourself? (thoughts of self-injury) = Yes]	72 (10.45%)	617 (89.55%)	1	**< 0.001**	0.345	0.230	0.519
	[@9. Ever had the idea of hurting yourself? (thoughts of self-injury) = No]	264 (16.59%)	1327 (83.41%)	0	. NA	. NA	. NA	. NA
	[@10 Whether affected by relationship factors such as classmates, parents, or teachers? = Yes]	230 (12.32%)	1637 (87.68%)	1	0.239	0.673	0.347	1.302
	[@10 Whether affected by relationships factors such as, classmates, parents, or teachers? = No]	106 (25.67%)	307 (74.33%)	0	. NA	. NA	. NA	. NA
	[@11 Coping style = Neutral]	23 (37.10%)	39 (62.90%)	1	**0.009**	0.388	0.190	0.793
	[@11 Coping style = Positive]	267 (12.62%)	1848 (87.38%)	1	**< 0.001**	0.256	0.164	0.401
	[@11 Coping style = Negative]	46 (44.66%)	57 (55.34%)	0	NA	NA	NA	NA

P<0.05 (bold values) indicated a significant multi-factor-correlation, and P<0.01(bold values) indicated a highly significant multi-factor-correlation for deperssed or non-depressed.

**Table 4:** Factors related to the depression level of participants aged 14–17 years.

coefficient a					
Model	Unstandardized Coefficients		Standardized Coefficients	T	P Value
	B	SE	Beta		
(constant)	2.026	0.050		40.661	**< 0.001**
Whether getting enough sleep?	0.088	0.012	0.158	7.378	**< 0.001**
Whether affected by relationship factors such as, classmates, parents, or teachers?	−0.096	0.021	−0.104	−4.689	**< 0.001**
Coping styles	−0.135	0.027	−0.102	−4.947	**< 0.001**
Ever had the idea of hurting yourself? (thoughts of self-injury)	−0.061	0.017	−0.079	−3.599	**< 0.001**
Family composition	0.017	0.006	0.057	2.813	**0.005**

a. Variables: 14–17-year-old groups: depressed and non-depressed P<0.01 (bold values) indicated the factor-model was highly significant for depressed and non-depressed.

Similarly, in the analysis of anxiety-related factors, unlike the predicted factors, eight factors were found to be related to the scores of the anxiety scale in the univariate analysis, with Ps less than 0.05 (family composition, parent/guardian care, regular sport participation, adequate sleep, previous depression, self-injurious thoughts, coping style, affected by interpersonal relationships; **Table 2**). In further multiple regression analyses, the influencing factors of anxiety were analyzed with age and gender used as covariates, and it was found that family composition was related to anxiety, with the nuclear family pattern not exhibiting protective effects for anxiety as predicted (P = 0.001 OR 1.517, 95% CI 1.192–1.931) and also related to the feeling that parents and elders care enough (P = 0.038 OR 1.840, 95% CI 1.034–3.273). Single-parent family (P = 0.030 OR 1.688, 95% CI 1.051–2.711) and reorganized family after parental divorce and remarriage (P = 0.040, OR 2.190, 95% CI 1.035–4.635) were both risk factors for anxiety. Feelings of sleep deprivation (P = 0.005, OR 0.651, 95% CI 0.482–0.879), having thoughts of self-injury (P < 0.001, OR 0.568, 95% CI 0.418–0.771), and feeling affected by classmates, parents, and teacher relationship factors (P = 0.016, OR 0.549, 95% CI 0.338–0.893) were associated with anxiety but were not risk factors for depression as predicted. Relatively neutral coping style (P < 0.001, OR 0.223, 95% CI 0.112–0.442) and positive coping style were protective factors for anxiety (P < 0.001, OR 0.352, 95% CI 0.228–0.545) (**Table 5**). In the analysis of anxiety-related factors, relationship factors, coping styles, family composition, self-injurious thoughts, and adequate sleep time were used to form a significantly correlated (all Ps less than 0.01) anxiety prediction model (**Table 6**).

**Table 5:** Multivariate regression analysis of anxiety in participants aged 14–17 years.

Intergroup analysis of anxiety for the 14–17-year-old group		Anxiety N (%)	Non-anxiety N (%)	df	P Value	Exp (B)	95% CI	
							low	up
1.0	intercept			1	0.470			
	@1. Gender: Male Female	365 (33.15%) 398 (33.76%)	736 (66.85%) 781 (66.24%)	1	0.068	1.192	0.987	1.440
	@2. Age: 14 years old 15 years old 16 years old 17 years old	31 (34.44%) 293 (32.96%) 240 (31.25%) 199 (37.34%)	59 (65.56%) 596 (67.04) 528 (68.75%) 334 (62.66%)	1	0.375	0.931	0.796	1.090
	[@3. Family composition = Nuclear family (children and parents)]	596 (35.10%)	1102 (64.89%)	1	**0.001**	1.517	1.192	1.931
	[@3. Family composition = Single parent family]	37 (37.37%)	62 (62.63%)	1	**0.030**	1.688	1.051	2.711
	[@3. Family composition = Reconstituted Family]	14 (42.42%)	19 (57.58%)	1	**0.040**	2.190	1.035	4.635
	[@3. Family composition = Large family: 3 generations]	116 (25.78%)	334 (74.22%)	0	NA	NA	NA.	NA
	[@4. Whether feeling parents or elders (caregivers) caring about yourself enough? = Yes]	747 (33.91%)	1456 (66.09%)	1	**0.038**	1.840	1.034	3.273
	[@4. Whether feeling parents or elders (caregivers) caring about yourself enough? = No]	16 (20.78%)	61 (79.22%)	0	Na	NA	NA	NA
	[@5. Whether participating in sports regularly? = Often]	370 (37.60%)	614 (62.40%)	1	0.660	1.069	0.793	1.441
	[@5. Whether participating in sports regularly? = Occasionally]	300 (29.64%)	712 (70.36%)	1	0.132	0.798	0.596	1.070
	[@5. Whether participating in sports regularly? = Hardly]	93 (32.75%)	191 (67.25%)	0	NA	NA	NA	NA
	[@7. Whether getting sleep is enough? = Sufficient]	348 (40.99%)	501 (59.01%)	1	0.573	1.098	0.794	1.517
	[@7. Whether getting sleep is enough? = Insufficient]	325 (27.31%)	865 (72.69%)	1	**0.005**	0.651	0.482	0.879
	[@7. Whether getting sleep is enough? = Seriously insufficient]	90 (37.34%)	151 (62.66%)	0	NA	NA	NA	NA
	[@8. Have ever been depressed? = Yes]	548 (30.63%)	1241 (69.37%)	1	0.389	1.216	0.779	1.896
	[@8. Have ever been depressed? = No]	215 (43.79%)	276 (56.21%)	0	NA	NA	NA	NA
	[@9. Ever had the idea of hurting yourself? (thoughts of self-injury) = Yes]	177 (25.69%)	512 (74.31%)	1	**< 0.001**	0.568	0.418	0.771
	[@9 .Ever had the idea of hurting yourself? (thoughts of self-injury) = No]	586 (36.83%)	1005 (63.17%)	0	NA	NA	NA	NA
	[@10 Whether affected by factors such as study pressure, peer relationships, parents, teachers, etc? = Yes]	569 (30.48%)	1298 (69.52%)	1	**0.016**	0.549	0.338	0.893
	[@10 Whether affected by factors such as study pressure, peer relationships, parents, teachers, etc? = No]	194 (46.97%)	219 (53.03%)	0	NA	NA	NA	NA
	[@11 Coping styles=neutral]	27 (43.55%)	35 (56.45%)	1	**< 0.001**	0.223	0.112	0.442
	[@11 Coping styles = positive]	670 (31.68%)	1445 (68.32%)	1	**< 0.001**	0.352	0.228	0.545
	[@11 Coping styles=negative]	66 (64.08%)	37 (35.92%)	0	NA	NA	NA	NA

P<0.05 (bold values) indicated a significant multi-factor-correlation, and P<0.01 (bold values) indicated a highly significant multi-factor-correlation for anxiety or non-anxiety.

**Table 6:** Factors related to the anxiety level of participants aged 14–17 years.

coefficient a					
Model	Unstandardized Coefficients		Standardized Coefficients	T	P Value
	B	SE	Beta		
(constant)	2.043	0.067	NA	30.581	**< 0.001**
Whether affected by relationship factors such as, classmates, parents, and teachers?	−0.134	0.027	−0.110	−4.885	**< 0.001**
Coping styles	−0.196	0.037	−0.112	−5.372	**< 0.001**
Family composition	0.028	0.008	0.071	3.447	**0.001**
Ever had the idea of hurting yourself? (thoughts of self-injury)	−0.094	0.023	−0.091	−4.151	**< 0.001**
Whether getting enough sleep?	0.054	0.016	0.073	3.391	**0.001**

a. Variables: 14–17-year-old groups: anxiety and non-anxiety groups. P<0.01 (bold values) indicated that factor-model was highly significant for anxiety and non-anxiety.

In the 18–25-year-old group (N = 395), the incidence of depression and anxiety was low, 34 (8.6%) and 7 (1.8%), respectively. In the T-test/ANOVA, only whether had depressed mood or not was an influencing factor for anxiety score (F = 4.920, P = 0.027; **Table 7**). Chi-square analysis and a linear model analysis based on age and gender factors showed that only depression was a significant factor for anxiety (P < 0.05), which could be used as a predictor of anxiety (**Table 8**).

**Table 7:** T-test/ANOVA of anxiety and depression scores in participants aged 18–25 years.

Variables N (%)			The anxiety score of the 18–25-year-old group			The depression score of the 18–25-year-old group		
			(X ± s)	T/F	P	(X ± s)	T/F	P
Gender	male female	94 (23.80%) 301 (76.20%)	34.55 ± 6.36 33.43 ± 7.32	1.773	0.184	35.35 ± 9.93 34.07 ± 9.43	1.288	0.257
Age	14–17 years 18–25 years	373 (94.43%) 22 (5.57%)	33.64 ± 7.14 33.72 ± 6.76	0.478	0.490	34.31 ± 9.61 35.40 ± 8.69	0.269	0.604
Family composition	Nuclear family (children and parents) Single parent family Reconstituted Family Large family: 3 generations	261 (66.08%) 36 (9.11%) 11 (2.78%) 87 (22.03%)	33.63 ± 7.31 34.06 ± 5.33 31.36 ± 4.63 34.05 ± 7.42	0.505	0.679	34.45 ± 9.61 34.06 ± 7.42 32.73 ± 6.12 34.47 ± 10.56	0.129	0.943
Are you an only child?	Yes No	213 (53.92%) 182 (46.08%)	33.76 ± 7.63 33.63 ± 6.48	0.032	0.857	34.69 ± 10.13 34.00 ± 8.84	0.514	0.474
Do you feel like your parents (or caregivers/elders) care enough about you?	Yes No	381 (96.46%) 14 (3.54)	33.71 ± 7.17 33.30 ± 5.43	0.044	0.834	34.36 ± 9.59 34.64 ± 8.71	0.012	0.914
Does your family struggle financially?	Yes No	79 (19.24%) 316 (80.76%)	33.96 ± 7.16 33.63 ± 7.11	0.131	0.717	35.32 ± 10.01 34.13 ± 9.43	0.969	0.326
Are you satisfied with the current state of your life?	Satisfied General Dissatisfied	242 (61.27%) 138 (34.94%) 15 (3.80%)	33.53 ± 6.97 34.08 ± 7.57 32.92 ± 4.76	32.92	4.76	34.14 ± 9.19 34.92 ± 10.50 33.00 ± 5.40	0.449	0.638
Do you regularly participate in sports?	Often Occasionally Hardly	87 (22.03%) 210 (53.16%) 98 (24.81%)	34.05 ± 8.43 33.51 ± 6.89 33.78 ± 6.32	0.185	0.832	34.80 ± 11.01 34.30 ± 9.27 34.13 ± 8.81	0.122	0.885
Do you enjoy online activities such as gaming or online dating?	Yes No	226 (57.22%) 169 (42.78%)	33.84 ± 7.35 33.51 ± 6.80	0.211	0.646	34.75 ± 10.04 33.86 ± 8.86	0.839	0.360
Do you spend a large amount of time on the Internet each day?	Yes No	232 (58.73%) 163 (41.27%)	33.66 ± 7.02 33.75 ± 7.26	0.016	0.900	34.30 ± 9.45 34.47 ± 9.73	0.031	0.861
Are you getting enough sleep?	Sufficient Insufficient Seriously insufficient	222 (56.20%) 146 (36.96%) 27 (6.84%)	33.02 ± 6.97 34.58 ± 7.02 34.44 ± 8.39	2.286	0.103	34.08 ± 9.46 34.69 ± 9.44 35.00 ± 11.10	0.241	0.786
Have you ever been depressed?	Yes No	292 (73.92%) 103 (26.08%)	34.17 ± 7.33 32.37 ± 6.29	4.920	**0.027**	34.66 ± 9.71 33.54 ± 9.07	1.044	0.308
Have you ever had thoughts about hurting yourself (thoughts of self-injury)?	Yes No	0 395 (100%)	NA NA	NA	NA	NA 34.37 ± 9.55	NA NA	NA NA
Have you ever done anything to hurt yourself (behaviors of self-injury)?	Yes No	4 (1.01%) 391 (98.99%)	36.25 ± 6.85 33.67 ± 7.12	0.520	0.471	38.44 ± 13.78 34.33 ± 9.51	0.732	0.393
Coping styles	Neutral Positive Negative	62 (15.70%) 301 (76.20%) 32 (8.10%)	32.16 ± 7.45 34.02 ± 7.01 33.59 ± 7.20	1.782	0.170	33.97 ± 10.66 34.66 ± 9.51 32.46 ± 7.42	0.827	0.438
Are you affected by interpersonal relationships, such as those with classmates, parents, or teachers?	Yes No	292 (73.92%) 103 (26.08%)	27.23 ± 5.61 26.19 ± 5.88	2.514	0.114	34.56 ± 9.37 33.82 ± 10.08	0.457	0.499

P<0.05 (bold values) indicated a significant single-factor-correlation, and P<0.01 (bold values) indicated a highly significant single-factor-correlation for anxiety score and depression score.

**Table 8:** Factors related to the anxiety level of participants aged 18–25 years.

coefficient a					
Model	Unstandardized Coefficients		Standardized Coefficients	Unstandardized Coefficients	P Value
	B	SE	B		
(constant)	1.773	0.045	NA	39.715	**<0.001**
Depression groups: Depressed and Non-depressed	0.109	0.023	0.232	4.738	**<0.001**

a. Variables: 18–25-year-old groups: anxiety and non-anxiety groups. P<0.01 (bold values) indicated that factor-model (depressed and non-depressed) was highly significant for anxiety and non-anxiety.

Finally, a Pearson’s correlation analysis of each research scale tool was performed, and a significant correlation between scale scores was observed (**Tables 9**, **10**). Additionally, a comparative analysis of depression and anxiety was conducted in participants aged 16–17 years, which found that, although the two scales were suitable for different age groups, there was no difference in the recognition of depression and anxiety, depression scale (K = 0.687, P = 0.403) and anxiety scale (K=2.505, P = 0.113).

**Table 9:** Pearson’s analysis of SCAS, CDI, Negative Coping Style, and Positive Coping Style scales in participants aged 14–17 years.

Variables		SCAS	CDI	Negative coping style score	Positive coping style score
SCAS	Pearson’s correlation	1	0.688^**^	0.206^**^	−0.306^**^
	P Value	NA	**<0.001**	**<0.001**	**<0.001**
	N	2280	2280	2280	2280
CDI	Pearson’s correlation	0.688^**^	1	0.202^**^	−0.435^**^
	P Value	**<0.001**	NA	**<0.001**	**<0.001**
	N	2280	2280	2280	2280
Negative coping style score	Pearson’s correlation	0.206^**^	0.202^**^	1	0.144^**^
	P Value	**<0.001**	**<0.001**	NA	**<0.001**
	N	2280	2280	2280	2280
Positive coping style score	Pearson’s correlation	−0.306^**^	−0.435^**^	0.144^**^	1
	P Value	**<0.001**	**<0.001**	**<0.001**	NA
	N	2280	2280	2280	2280

**P < 0.01. P<0.01 (bold values) indicated a highly significant Pearson’s correlation.

**Table 10:** Pearson’s analysis of SAS, SDS, positive Coping Style, and Negative Coping Style scales in participants aged 18–25 years.

Variables		Positive coping style score	Negative coping style score	SAS	SDS
Positive coping style score	Pearson’s correlation	1	0.716^**^	−0.027	−0.041
	P Value	NA	**<0.001**	0.586	0.412
	N	395	395	395	395
Negative coping style score	Pearson’s correlation	0.716^**^	1	−0.058	−0.056
	P Value	**<0.001**	NA	0.251	0.266
	N	395	395	395	395
SAS	Pearson’s correlation	−0.027	−0.058	1	0.643^**^
	P Value	0.586	0.251	NA	<0.001
	N	395	395	395	395
SDS	Pearson’s correlation	−0.041	−0.056	0.643^**^	1
	P Value	0.412	0.266	**<0.001**	NA
	N	395	395	395	395

**P < 0.01. P<0.01 (bold values) indicated a highly significant Pearson’s correlation.

## Discussion

More attention should be paid to the mental health of young groups, who face a world with multiple crises and much uncertainty. Most mental disorders become more prevalent in adolescence, but rare during childhood. The prevalence of suicidal ideation, planning, and attempts together represented the fourth leading cause of death for adolescents between age 15–19 years globally in 2019, although still relatively low in adolescents, and also presented both medical and societal challenges (20). A high incidence rate of depression has been observed in adolescents, with a national survey conducted by Grunbaum et al. finding 28.6% of high school students feeling sad or hopeless almost every day, causing them to stop some daily activities; 16.9% reporting having seriously considered suicide; 16.5% having planned suicide; and 8.5% reporting one or more suicide attempts (21). Therefore, there is little doubt that self-injury and suicide in adolescents are problems requiring immediate attention. In another previous study on adolescent depression in Huangshi, China during the COVID-19 pandemic, depression was the most frequently observed mental illness in 674 patients observed (282, 41.84%), with the age ranging from 9 to 18 years, and most of the subjects were in high school (261/282, 92.55%). Genetic factors, school violence, academic stress, sleep disturbances, and family-related factors are important factors that contribute to depression, self-injury, and even suicide or suicide attempts (22). In this study, among adolescents aged 14–17 years, the incidence of depression and anxiety was 336 (14.7%) and 763 (33.5%), respectively. Among subjects aged 18–25 years, the incidence of depression and anxiety was 34 (8.6%) and 7 (1.8%), respectively. It was also observed that most subjects with depression and anxiety were in high school. Among the total sample, 2081 (77.8%) subjects reported having depression at some point in their life, while 689 (25.8%) and 323 (12.1%) subjects reported thoughts and behaviors of self-harm, respectively. While depression and anxiety both showed elevated levels in the current study, which certainly deserves attention.

However, in the current study, an analysis of factors influencing depression in adolescents aged 14–17 years showed that age (P = 0.000, OR 0.661, 95% CI 0.5370.813) was a protective factor, but the nuclear family structure (parents and children) was significantly associated with depression (p = 0.047, OR 1.411, 95% CI 1.004–1.983) and may not be a protective factor as predicted. This suggests that parental presence does not absolutely and universally improve children’s emotional state. This may be similar to the conclusion of previous studies that found depression is related to specific parenting styles, rather than simple family composition. Analysis of the factors that influence anxiety showed family composition was related to anxiety and that the nuclear family pattern was also not a protective factor for anxiety as predicted (P = 0.001, OR 1.517, 95% CI 1.192–1.931), while a single-parent and restructured family structures were risk factors (P = 0.030, OR 1.688, 95% CI 1.051–2.711; P = 0.040, OR 2.190, 95% CI 1.035–4.635, respectively). Further, feeling that parents and elders exhibited enough concern was not a protective factor for anxiety as predicted (P = 0.038 , OR 1.840, 95% CI 1.034–3.273). Therefore, family environmental factors are also important factors that influence adolescents’ emotional state. However, whether it is a positive impact or a negative impact depends on the actual situation, which requires further research.

At present, the factors of depression in adolescents and early adulthood are multifaceted, from environmental to specific events. Future research on adolescent depression also needs to explore the effects of science and technology networks; for example, recent studies have found that cultural tension caused by social media may cause unnecessary risk to adolescents and other sleep-deprived people, who sometimes become dependent on social media to kill time, and that this time spent online is strongly associated with the occurrence of depression (23). In this study, T-test/ANOVA found that the time spent on the Internet was a factor that influenced depression (F = 11.161, P = 0.001), but only in adolescents aged 14–17 years. While a statistical difference was observed between depressed and non-depressed groups, no such association was observed between anxiety groups, suggesting that the impact of online games may be greater in younger adolescents. Whether it is better to be exposed to In review 12 the Internet and games in adulthood to reduce the occurrence of negative emotions requiring further research.

In addition, the emotional state of some adolescents was shown to be related to interpersonal relationships. Adolescence is characterized by increased peer interactions and increased sensitivity to peer assessment, which may include classmate and teacher evaluations. Previous studies have shown that poor social relationships are associated with the occurrence of depression (24). Good social relationships with parents and peers can protect children and adolescents from developing mental disorders in adulthood, while some negative experiences can increase the risk of depression later in life. Among them, adolescent bullying victimization was the strongest predictor of depression in all children, with childhood loneliness also associated. However, spending time with family seems to prevent the negative effects of bullying and loneliness. Online social evaluations can have certain impacts on adolescents and may be related to negative adolescent emotions, especially when the individual feels threatened by social evaluations (25). In this study, the relationship between participants and classmates, family, and teachers was also investigated. In adolescents aged 14–17 years, the presence of any of the above relationships was related to depression and anxiety scores, which was a potential influencing factor for the occurrence of depression and anxiety (P all < 0.001). In the multivariate and linear model analyses, relationships, perceived adequacy of sleep, coping styles, thoughts of self-injury, and family composition gradually became a prediction model of depression, with significant differences observed (all Ps less than 0.05). In the analysis of anxiety-related factors, the relationships, coping style, family composition, thoughts of self-injury, and perceived sleep adequacy became a prediction model of anxiety, which were significantly correlated (all Ps less than 0.01). In addition, 95.18% (1777/1867) of the 14–17-year-olds who reported depression and 99.66% (291/292) of the 18–25-year-olds who reported depression believed that the above relationship was related. Although a number of factors have not allowed for a more detailed investigation, this at least suggests that the occurrence of depression is related to the circumstances of these relationships. This emphasizes the need to pay attention to the emotional changes of adolescents while also paying attention to the impact of these relationships and the importance of family companionship in daily life. Moreover, it is necessary to pay attention to how these relationships form, both in-person and online. As such, the negative impact of Internet use on adolescents may not be from playing games but because of negative evaluation. More studies on the specific impact of online social relationships and evaluation on adolescent emotional state are needed in the future. In this study, coping styles were potential influencing factors of depression and anxiety scores in adolescents aged 14–17 years (F = 64.440, P < 0.001; F = 33.570, P < 0.001, respectively). Neutral and positive coping styles were protective factors for depression (P = 0.009, OR 0.388, 95% CI 0.190–0.793; P < 0.001, OR 0.256, 95% CI 0.164–0.401, respectively). Relatively neutral coping styles (P < 0.001, OR 0.223, 95% CI 0.112–0.442) and positive coping styles (P < 0.001, OR 0.352, 95% CI 0.228–0.545) were protective factors for anxiety. For behavioral patterns, in this study, those who took a negative approach were more likely to be depressed, so actively adjusting coping styles and cultivating the cognitive model of children and adolescents may help to prevent depression. Studies have shown that stress is a risk factor for unipolar and bipolar disorders, but behavioral responses to stress, such as impulsivity and social withdrawal, may mediate the association between stress and specific mood symptoms. Different response patterns distinguish subgroups of people who are susceptible to specific types of emotional symptoms (26). These findings suggest that having an external locus of control and holding negative cognitive styles in mid- to late-adolescence are associated with an increased likelihood of probable depression in young adulthood (27). These studies show that behavioral responses to stress, including coping styles, are important aspects that should be paid attention to, as coping styles have an important impact on emotional state, which is consistent with the results of this study. However, a number of factors may also have mediated the final outcome. Longitudinal studies have suggested that self-regulation and shame may mediate the relationships among ACEs, positive childhood experiences (PCEs), and adolescent health. PCEs negatively predict stigma and positively predict self-regulation, while ACEs negatively predict self-regulation. Self-regulation mediates the relationship between ACEs or PCEs and anxiety and also the relationship between ACEs and substance abuse. Self-regulation appears to be particularly important in preventing depression, anxiety, and substance abuse in early adulthood (28). Therefore, the ability to adjust is very important. It is always necessary to find appropriate and effective interventions according to the cause; understanding adolescents’ self-regulation and coping styles is helpful for understanding the occurrence of depression and anxiety and for formulating coping measures. These findings highlight not only the diversity and complexity of factors that contribute to the occurrence of depression and anxiety in adolescents but also the fact that early identification is needed. On the basis of these studies, prevention and intervention measures are further proposed, which help to determine the specific form and timing of the implementation of interventions (29, 30). These are still subjects for follow-up studies.

## Limitations

While the findings of the current study are important, there are several limitations that should be considered. First, the sample size was small and all the students were from the same geographic area. This was particularly true of the early adulthood sample, which may cause a certain deviation in the results. Second, the detection of related factors, specifically the analysis of stress and relationship factors, was not comprehensive. The results show that depression and anxiety were related to relationships with various entities, including classmates, teachers, and families in the 14–17-year-old group. However, no further in-depth investigation was conducted in the current study, such as specific event types and ways of interacting. Follow-up research on social relationships is needed to explore this issue in depth. Third, although the results of this study showed that gender was not a statistically significant factor in both age groups, it was an influencing factor for depression in 14–17-year-olds in the univariate analysis (F = 5.015, P = 0.025); however, it is unclear whether other gender-related factors, such as sexual orientation, play an important role in adolescent emotional state. In fact, longitudinal studies on depression trajectories have shown that sexual minorities have a higher incidence of depression among adolescents, and these differences can persist from early adolescence to early adulthood (31). Unfortunately, in the current study, sexual orientation was not included in the survey factors due to the group characteristics of the study subjects. Although the sexual minority group likely only accounted for a small proportion of the overall group, it may have been an important factor affecting the emotional state of adolescents and early adults. Fourth, this study did not conduct a dimensional categorical analysis of the specific symptoms of depression and anxiety, considering the youthful attributes of the study population, and it did not conduct a systematic investigation and correlation analysis of cognitive function, even though other studies have shown that unipolar depression and bipolar depression in adolescence and early adulthood are associated with impaired executive functioning (32). Finally, depression and anxiety may be related to other factors that were not considered in this study, due to the characteristics of the study sample. It is recommended that further investigations should be conducted in the adolescent populations in the future.

## Conclusion

The incidence of depression and self-injury in adolescents and young adults was high, with 2081 (77.8%) reporting depression at some point of their life, 689 (25.8%) reporting thoughts of self-injury, and 323 (12.1%) reporting having engaged in self-injurious behaviors. Specific attention needs to be paid to the adolescent population (14–17-year-olds). Among this group, 1789 subjects (78.46%) reported being depressed at some point of their life, 689 (30.22%) reported having thoughts of self-injury, and 319 (1.71%) reported engaging in self-injury behaviors, with the incidence of active depression, anxiety, and comorbid depression/anxiety observed at 336 (14.7%), 763 (33.5%), and 273 (11.97%), respectively. Age is still an important factor in the occurrence of depression and anxiety, as the incidence of depression and anxiety was lower in the 18–25-year-old group when compared to that in the 14–17-year-old group (8.6% vs. 14.7% and 1.8% vs. 33.5%). In the analysis of risk factors for depression and anxiety, the relationship with classmates, teachers, and parents was still an important factor influencing the emotional state, and good coping style was found to be a protective factor for these diseases. In particular, the focus of prevention and intervention in the future should remain on the improvement of various relationships and the honing of coping skills.

## Data availability statement

The raw data supporting the conclusions of this article will be made available by the authors, without undue reservation.

## Ethics statement

The studies involving humans were approved by the Ethics Committee of the People’s Hospital of Liaoning Province. The studies were conducted in accordance with the local legislation and institutional requirements. Written informed consent for participation in this study was provided by the participants’ legal guardians/next of kin.

## Author contributions

XY: Conceptualization, Funding acquisition, Methodology, Project administration, Supervision, Writing – original draft, Writing – review & editing, Formal analysis. JM: Data curation, Investigation, Methodology, Software, Writing – original draft. YB: Data curation, Investigation, Methodology, Writing – review & editing. LL: Data curation, Investigation, Writing – original draft. GZ: Data curation, Formal analysis, Writing – review & editing.
